# Understanding the causes of errors in eukaryotic protein-coding gene prediction: a case study of primate proteomes

**DOI:** 10.1186/s12859-020-03855-1

**Published:** 2020-11-10

**Authors:** Corentin Meyer, Nicolas Scalzitti, Anne Jeannin-Girardon, Pierre Collet, Olivier Poch, Julie D. Thompson

**Affiliations:** Department of Computer Science, ICube, CNRS, University of Strasbourg, Strasbourg, France

**Keywords:** Genome annotation, Primates, Gene prediction, Protein sequence errors, Error correction

## Abstract

**Background:**

Recent advances in sequencing technologies have led to an explosion in the number of genomes available, but accurate genome annotation remains a major challenge. The prediction of protein-coding genes in eukaryotic genomes is especially problematic, due to their complex exon–intron structures. Even the best eukaryotic gene prediction algorithms can make serious errors that will significantly affect subsequent analyses.

**Results:**

We first investigated the prevalence of gene prediction errors in a large set of 176,478 proteins from ten primate proteomes available in public databases. Using the well-studied human proteins as a reference, a total of 82,305 potential errors were detected, including 44,001 deletions, 27,289 insertions and 11,015 mismatched segments where part of the correct protein sequence is replaced with an alternative erroneous sequence. We then focused on the mismatched sequence errors that cause particular problems for downstream applications. A detailed characterization allowed us to identify the potential causes for the gene misprediction in approximately half (5446) of these cases. As a proof-of-concept, we also developed a simple method which allowed us to propose improved sequences for 603 primate proteins.

**Conclusions:**

Gene prediction errors in primate proteomes affect up to 50% of the sequences. Major causes of errors include undetermined genome regions, genome sequencing or assembly issues, and limitations in the models used to represent gene exon–intron structures. Nevertheless, existing genome sequences can still be exploited to improve protein sequence quality. Perspectives of the work include the characterization of other types of gene prediction errors, as well as the development of a more comprehensive algorithm for protein sequence error correction.

## Background

An unprecedented number of genomes are being sequenced, offering a unique view of the specific characteristics of individual organisms and new opportunities to analyze life on a larger scale. An essential first step in the genome annotation process is the identification of all the coding regions of the genome. However, discovering genes in the new genome assemblies is challenging, especially in eukaryotes where the aim is to establish accurate gene models with precise exon–intron structures of all genes [1, 2]. While model organisms such as human or mouse are well-studied and experimental evidence is available for many genes [3–5], many sequences for non-model organisms are predicted by computational pipelines and may thus be incomplete or incorrect [6–8]. Furthermore, erroneous gene predictions are often propagated across genomes by homology-based genome annotation strategies. As a result, nucleotide and protein repositories, such as UniProt [9], RefSeq [10] or Ensembl [11], contain an increasing number of sequence errors or inaccuracies [12, 13].

Commonly encountered errors include missed proteins [14, 15], wrong exon and gene boundaries [16], non-coding nucleotide sequence retention in coding exons [17], as well as fragmentation or fusion of gene models [18]. These errors can be propagated in downstream applications, leading to incoherent or incorrect conclusions [19, 20]. Errors can severely affect studies of individual proteins, including transcript quantification, phylogenetic tree inference, and protein structure or function predictions. It has also been shown that gene prediction errors can lead to biases in large-scale statistical studies [19, 21, 22]. Therefore, DNA and protein sequence quality is essential and sequence information needs to be accurate, reliable, and accessible in a clear and consistent manner [23, 24].

Various strategies have been developed to help identify and correct errors in gene annotations, using information from comparative or evolutionary analyses [25–27], experimental evidence from expressed sequence tags (ESTs) or RNA-sequencing (RNA-seq) [22, 28], or dedicated sequence datasets such as the G3PO benchmark [29]. These studies generally agree that up to 50% of the protein sequences in the public databases have an least one error. However, very few studies have attempted to identify the underlying reasons for the badly predicted sequences.

**Fig. 1:**
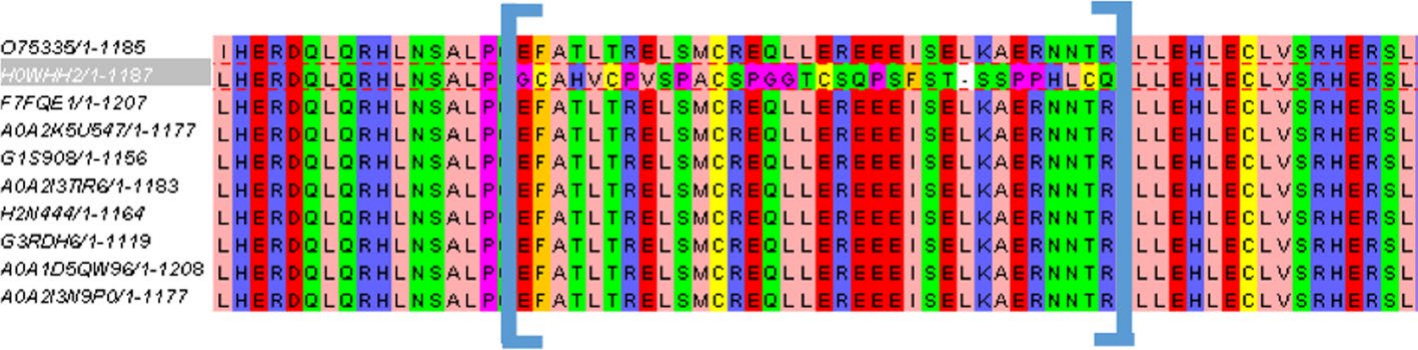
Part of the MSA of the Human Liprin-Alpha-4 protein (first from top) with orthologous proteins in nine other primates. The sequence from *Otolemur garnettii* (H0WHH2) contains a ‘mismatched segment’ in the region highlighted by blue square brackets

Here, we use a large set of 176,478 proteins from a representative set of ten primates in order to characterize different types of gene prediction errors and investigate their causes. The protein sequences were downloaded from the Uniprot reference proteomes and RefSeq databases. To identify potential sequence errors, including insertions, deletions and mismatched segments, we compared the primate sequences to the canonical isoforms of all known human proteins in the UniProt database. The UniProt canonical sequence for a given human protein generally corresponds to the most frequent or most conserved protein isoform in orthologous species. We then focused on the primate protein sequences containing mismatched segment errors, i.e. where part of the correct protein sequence is replaced with an alternative erroneous sequence, as shown in the example in Fig. 1. These errors cause particular problems for downstream applications, since the badly predicted amino acids can significantly affect structural and functional annotations, as well as conservation and phylogenetic analyses.

## Results

### Identification of sequence errors in primate proteins

**Fig. 2:**
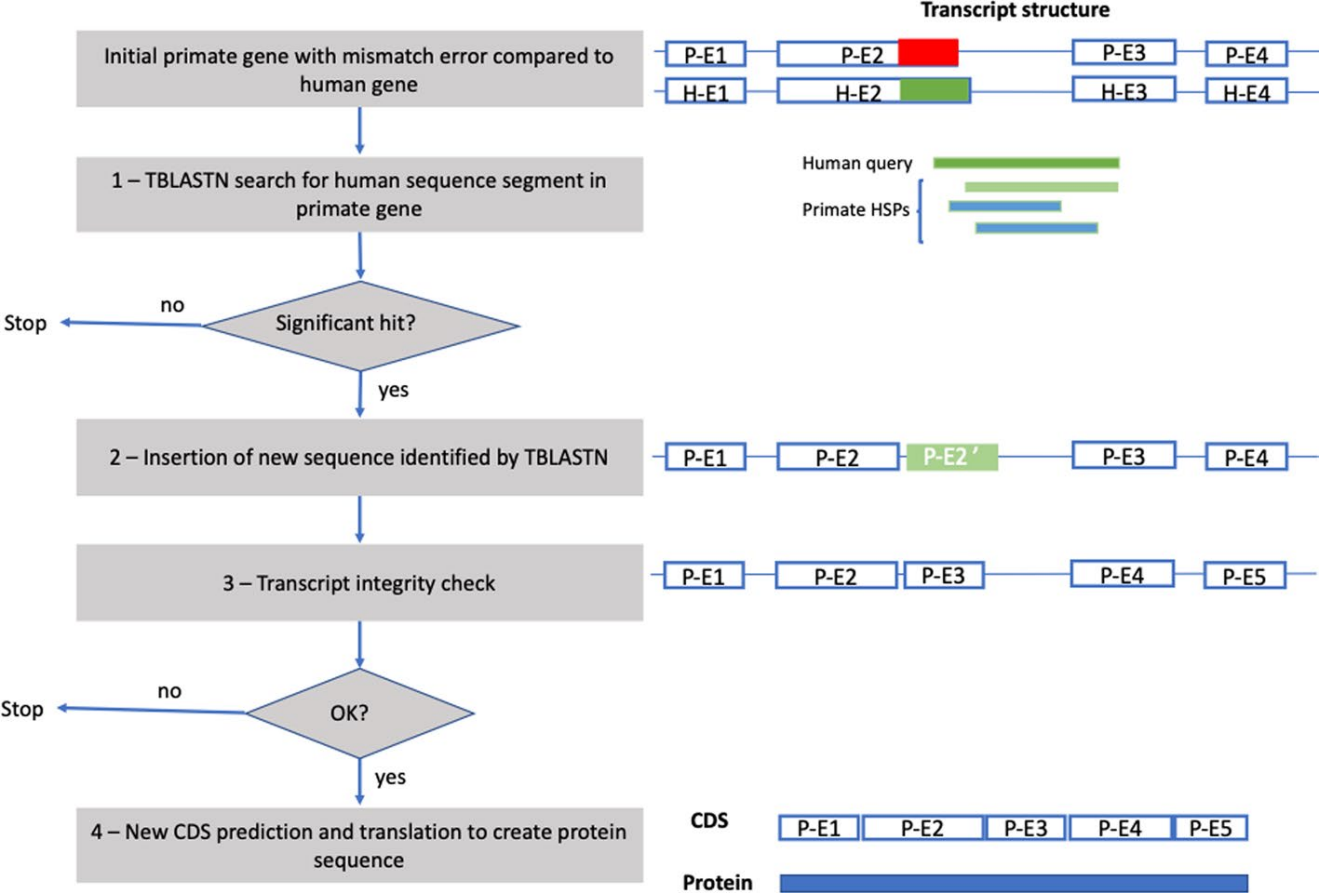
Schema of transcript correction protocol. Blue boxes represent exons and are labeled P-E(*i*) for primate exon number *i* and H-E(*i*) for human exon number *i*. Horizontal thin blue lines represent introns. Red box represents a primate sequence segment coding for a mismatch, compared to the human sequence (dark green box). Light green box represents a significant TBLASTN hit or high-scoring segment pair (HSP), which will be inserted in the primate gene as a new exon

To estimate the frequency of gene prediction errors, a large set of primate protein sequences was extracted from the Uniprot reference proteome database. The human proteome was used as a reference, since the proteins have been very widely studied and for the purposes of this study, are assumed to be accurate. By comparing closely related primate sequences with the human proteins, we could identify potential gene prediction errors in the other primate proteomes (Fig. 2). Orthologous relationships were predicted using BLASTP searches, where each human protein sequence was used as a query to search each primate proteome. Starting with the 20,595 human proteins, we thus obtained between 14,000 and 19,000 orthologous protein sequences for each primate (Table 1). In total, 176,478 orthologous proteins were retrieved from the primate proteomes, with an average of 85.7% of the human sequences found in each primate.

**Table 1:** Primate proteomes, number of orthologous proteins and human orthology rates

Organism	TAXID	Uniprot proteome	No. of predicted orthologs	% Human sequences with a primate ortholog
*Homo sapiens* (Human)	9606	UP000005640	20,595	100.0
*Pan Troglodytes* (Chimpanzee)	9598	UP000002277	19,010	92.3
*Gorilla gorilla gorilla* (Gorilla)	9595	UP000001519	18,540	90.0
*Macaca mulatta* (Macaque)	9544	UP000006718	18,327	89.0
*Macaca fascicularis* (Crab-eating macaque)	9541	UP000233100	17,976	87.3
*Chlorocebus Sabaeus* (Vervet-AGM)	60,711	UP000029965	17,948	87.1
*Papio Anubis* (Olive baboon)	9555	UP000028761	17,904	86.9
*Pongo abelii* (Orangutan)	9601	UP000001595	17,814	86.5
*Nomascus leucogenys* (Gibbon)	61,853	UP000001073	17,478	84.9
*Callithrix jacchus* (Marmoset)	9483	UP000008225	17,110	83.1
*Otolemur garnettii* (Bushbaby)	30,611	UP000005225	14,371	69.8

Potential gene prediction errors were detected based on the MSAs of the orthologous proteins. For the 176,478 primate sequences extracted from Uniprot, a total of 82,305 sequence errors were identified, including insertions, deletions and mismatched segments (Table 2). In other words, 47% of the protein sequences are expected to have at least one error in agreement with previous studies. Internal deletions were the most commonly detected errors (29,045), followed by internal insertions (12,436) and mismatched segments (11,015). More errors were detected at the N-terminus than at the C-terminus for both sequence extensions (10,280 and 4573, respectively) and deletions (10,264 and 4672, respectively), although this could be due at least in part to the error detection algorithm used.

**Table 2:** Number of protein sequence errors detected in Uniprot primate sequences, for each of the error types

Primate	N-terminal extension	N-terminal deletion	C-terminal extension	C-terminal deletion	Internal insertion	Internal deletion	Mismatched segment	Total errors
*Callithrix jacchus*	1427	398	532	274	1315	1593	694	6233
*Chlorocebus Sabaeus*	914	1870	480	766	1840	2901	992	9763
*Gorilla gorilla gorilla*	820	1104	389	392	828	3211	1043	7787
*Macaca fascicularis*	918	667	448	295	964	2016	703	6011
*Macaca mulatta*	1657	402	661	235	1702	1403	561	6621
*Nomascus leucogenys*	757	1446	478	554	1186	6744	2641	13,806
*Otolemur garnettii*	603	1879	271	682	1417	2352	1887	9091
*Papio Anubis*	1134	434	500	286	1108	2342	1091	6895
*Pongo abelii*	1183	1673	370	981	1280	4877	802	11,166
*Pan troglodytes*	867	391	444	227	796	1606	601	4932
*TOTAL*	10,280	10,264	4573	4692	12,436	29,045	11,015	82,305

**Fig. 3:**
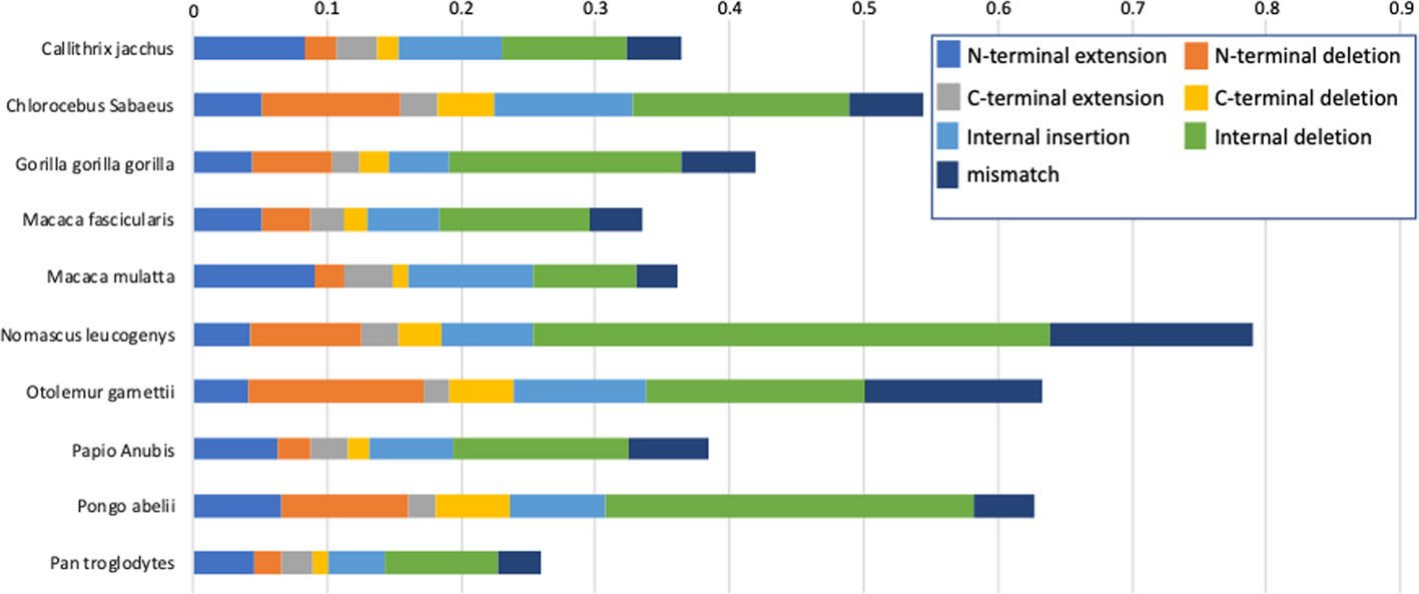
Frequency of gene prediction errors per protein sequence, in each of the Uniprot primate proteomes

As shown in Fig. 3, the frequency of gene prediction errors (normalized by the number of orthologous proteins detected) is not the same for all primate proteomes tested. While all the primates have globally similar distributions of the seven types of protein sequence error, some proteomes had significantly more errors than others. The number of errors per sequence ranges from 0.26 for *P. troglodytes* proteins to 0.79 for *N. leucogenys* proteins.

### Mismatch gene prediction errors

We then focused our analyses on the 11,015 potential mismatch sequence errors, as these cause specific problems for subsequent analyses. Mismatches were identified in all of the primate proteomes, with the lowest number of mismatches occurring in the *M. mulatta* protein sequences (561 mismatches, or 0.03 per protein) and the highest number of mismatches in the *N. leucogenys* sequences (2641 mismatches, or 0.15 per protein).

**Fig. 4:**
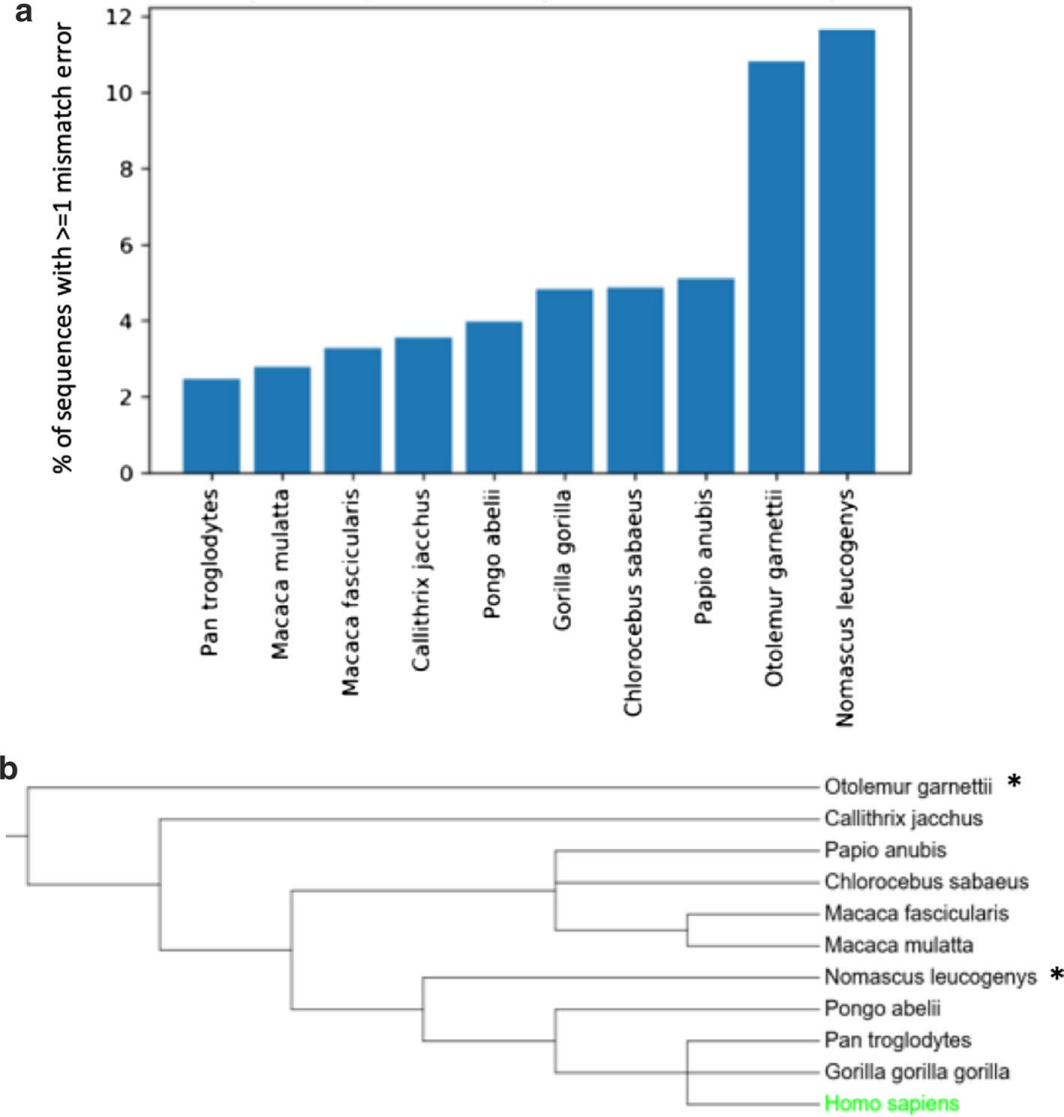
Comparison of primate sequence error rates and evolutionary distance from the human reference proteome. **a** Percentage of sequences with at least one mismatched segment for each primate. **b** Phylogenetic tree of the 11 primates included in the study, including the reference *Homo sapiens*. Asterisks indicate the species with high protein sequence error rates

To determine whether the mismatch error rate was linked to the evolutionary distance of the primates from the human reference, the percentage of sequences with at least one mismatch error was calculated for each primate proteome (Fig. 4a). While most of the primates have a mismatch rate between 2 and 5%, we identified two primates (*O. garnettii, N. leucogenys*) with as many as 11–12% of their sequences containing at least one mismatch. Surprisingly, the primate showing the highest mismatch rate (*N. leucogenys*) is not the most distant from human according to the tree of life (Fig. 4b). Furthermore, *G. g. gorilla*, which is very close to human in the tree of life, has a medium mismatch rate of 5.5%. These results taken together show that mismatch detection is not only dependent on the phylogenetic distance between primates and human, and there must be other factors to explain these observations.

Finally, to investigate whether the mismatch error rates were an issue related specifically to protein sequences from the Uniprot database, the same detection protocol was applied using the RefSeq protein database as the source for the primate reference proteomes. A similar number of orthologous proteins (181,223 primate proteins) were extracted from the RefSeq database, however 5971 mismatch errors were detected, compared to 11,015 for the Uniprot sequences. We conclude that mismatches are present in both databases, although the error rates seem to be lower in RefSeq than in Uniprot.

### Characterization of mismatch errors

To understand why mismatch errors were detected in the Uniprot protein sequences, each mismatch error was characterized using nine different features. These features can be divided into three categories: (i) features suggesting that our mismatch identification protocol identified a real gene misprediction, (ii) features suggesting that our mismatch identification protocol identified a false positive mismatch (for example, a mismatch resulting from a wrong ortholog prediction or a MSA error), and (iii) features that were uncertain (not clear whether the detected mismatch corresponds to a gene misprediction or a false positive). Out of 11,015 mismatches, 7401 (67.2%) could be associated with one or more features, leaving one third of the mismatches so far unexplained (Table 3).

**Table 3:** Characterization of 11,015 mismatched sequence segments in primate sequences, according to nine different features

Class	Feature	No. (%) of errors
Evidence of gene prediction error	Genomic sequence contains N characters (introns or exons)	5256 (47.7%)
	Primate sequence contains short introns (< 30 nucleotides)	937 (8.5%)
	1 Human exon aligned with ≥ 3 primate exons	611 (5.5%)
	Non-canonical splice sites in human sequence	237 (2.2%)
	Frameshift in primate exon sequence	138 (1.3%)
Evidence of false positive error	Human isoform exists that matches primate sequence	1194 (10.8%)
	Multiple alignment error	244 (2.2%)
	In a repeated protein region	232 (2.1%)
Mixed evidence	Mismatch associated with evidence of both gene prediction error and false positive error	341 (3.1%)
Unconfirmed	Conserved in ≥ 4 primates	1054 (9.6%)
	Mismatch associated with evidence of gene prediction error only	5446 (49.4%)
	Mismatch associated with evidence of false positive error only	4174 (37.9%)
	Mismatch associated with at least 1 feature	7401 (67.2%)
	Mismatch associated with 0 features	3614 (32.8%)

**Fig. 5:**
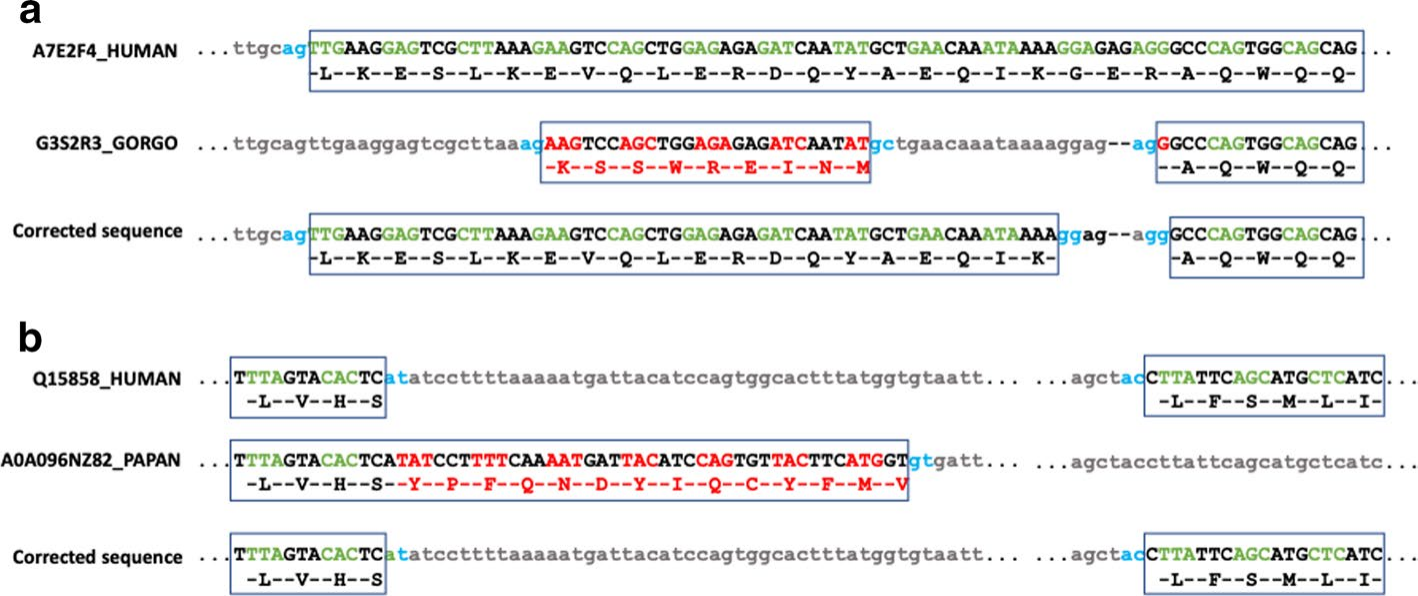
Example mismatched segments corresponding to gene prediction errors. **a** A deletion of 2 bases in the primate sequence G3S2R3 results in misprediction of 5′ part of exon. **b** Non-canonical splice sites in the human sequence Q15858 and misprediction of the primate sequence A0A096NZ82. Black boxes represent exons with nucleotide sequences shown in upper case, and protein sequences shown below the translated exon sequence. For the correctly predicted exons, alternate codons are shown in green. For the badly predicted exons, alternate codons are shown in red. Intron sequences are in lower case, with splice sites in blue

Almost half of the potential mismatches (5446 mismatches, 49.4%) were associated uniquely with evidence of a gene misprediction error (details are provided in Additional file 1). The most frequent feature associated with misprediction is the presence of undefined genomic regions (represented by N characters) in introns or exons, which were found in 47.7% of all mismatches. Introns shorter than the minimum length of 30 nucleotides required for a functional intron [30], are found in 8.5% of all mismatches. The presence of short introns is also sometimes linked to the presence of several small primate exons, corresponding to a single exon in the human gene (5.5%). Figure 5a shows an example of a badly predicted sequence from *G. gorilla* (G3S2R3_GORGO), which has an exon in the wrong frame compared to the human gene and an intron of length 21 nucleotides. A deletion of 2 bases compared to the exon of the human reference sequence A7F2E4_HUMAN (golgin A8 family member A) is probably due to a genome sequencing error. The sequence could be improved by creating an alternative longer exon in the correct reading frame, although this means introducing a short intron of only 7 nucleotides.

Other factors causing gene mispredictions include the presence of frameshifts in the primate exon sequences (1.3%) or the use of non-canonical splice sites in human sequences (2.2%). Figure 5b shows an example of a badly predicted primate sequence (A0A096NZ82_PAPAN) from *P. Anubis,* which is orthologous to the human sequence Q15858_HUMAN (Sodium channel protein type 9 subunit alpha) with non-canonical splice sites. A completely corrected primate sequence can be proposed if non-canonical splice sites are allowed in the gene model.

A number of false positive mismatches (37.9%) were found that could not be reliably classified as gene prediction errors. For 10.8% of all mismatches, an alternative human isoform could be found in the Ensembl database corresponding to the primate sequence. In this case, the mismatch error was probably caused by the definition of different canonical isoforms for human and primate sequences in the Uniprot database.

Finally, 12.7% of the mismatches were associated with at least one feature, but the cause of the error remains unconfirmed. These unconfirmed cases were mainly due to the detection of the mismatch in four or more primates (9.6% of all mismatches), which suggests that either the same misprediction was propagated across multiple primates or the human protein sequence is in fact significantly different from the non-human primates and the mismatch is a false positive. The remaining 3.1% of mismatches were associated with both misprediction evidence and false positive evidence and were also classed as ‘Unconfirmed’.

### Correction of mismatch errors

For the primate sequences containing mismatches linked to gene misprediction, we then tried to correct the mismatch errors using the correction protocol defined in the Methods section. The protocol first identifies the human sequence segment corresponding to the primate mismatch error and then uses the human segment to perform a TBLASTN search for a conserved segment in the primate genome. Out of 5446 mismatches associated with misprediction, refined sequences were proposed for 603 of them (~ 11%). The remaining mismatches could not be improved due to either a lack of significant TBLASTN hits, or issues with the integrity of the refined primate transcript.

To validate the proposed error corrections, the refined sequences were realigned with the human reference sequence and the error detection protocol was again performed on the full-length protein sequences. The number of sequence errors detected for the original and the refined proteins are shown in Table 4. A significant decrease in gene prediction errors can be observed, especially for the number of mismatched segments as might be expected. For the 603 proteins, 833 mismatch errors were detected in the original sequences, while only 263 were detected after refinement, i.e. 570 mismatch errors were fully corrected. The remaining mismatches (263) could not be corrected for several reasons: the refined sequence contains false positive mismatch errors due to MSA errors for example, or the correction protocol badly predicted the new sequence.

**Table 4:** Number of sequence errors identified before and after correction of 603 primate sequences

Error type	No. of errors before correction	No. of errors after correction	Difference
Internal insertion	340	132	− 208
Internal deletion	816	785	− 31
Mismatch	833	263	− 570
N-terminal insertion	32	32	0
N-terminal deletion	80	90	+ 10
C-terminal insertion	29	26	− 4
C-terminal deletion	54	52	− 2
TOTAL	2184	1379	− 805
Mean % identity	91.9%	95.0%	+ 3.1%
Mean % coverage	89.5%	90.7%	+ 1.2%

Interestingly, we also observed a decrease in the number of internal insertion errors, since the mismatch refinement could also affect nearby internal insertions. In contract, a small increase in the number of N-terminal deletions was observed, due to the new CDS prediction during the correction protocol. When the original start codon cannot be mapped onto the new transcript (for example, when the mismatch error occurs at the beginning of the protein), an alternative start codon is used, leading to possible N-terminal deletion errors. Taking into account all the error types, the correction protocol leads to a decrease of 37% in the number of detected sequence errors.

**Fig. 6:**
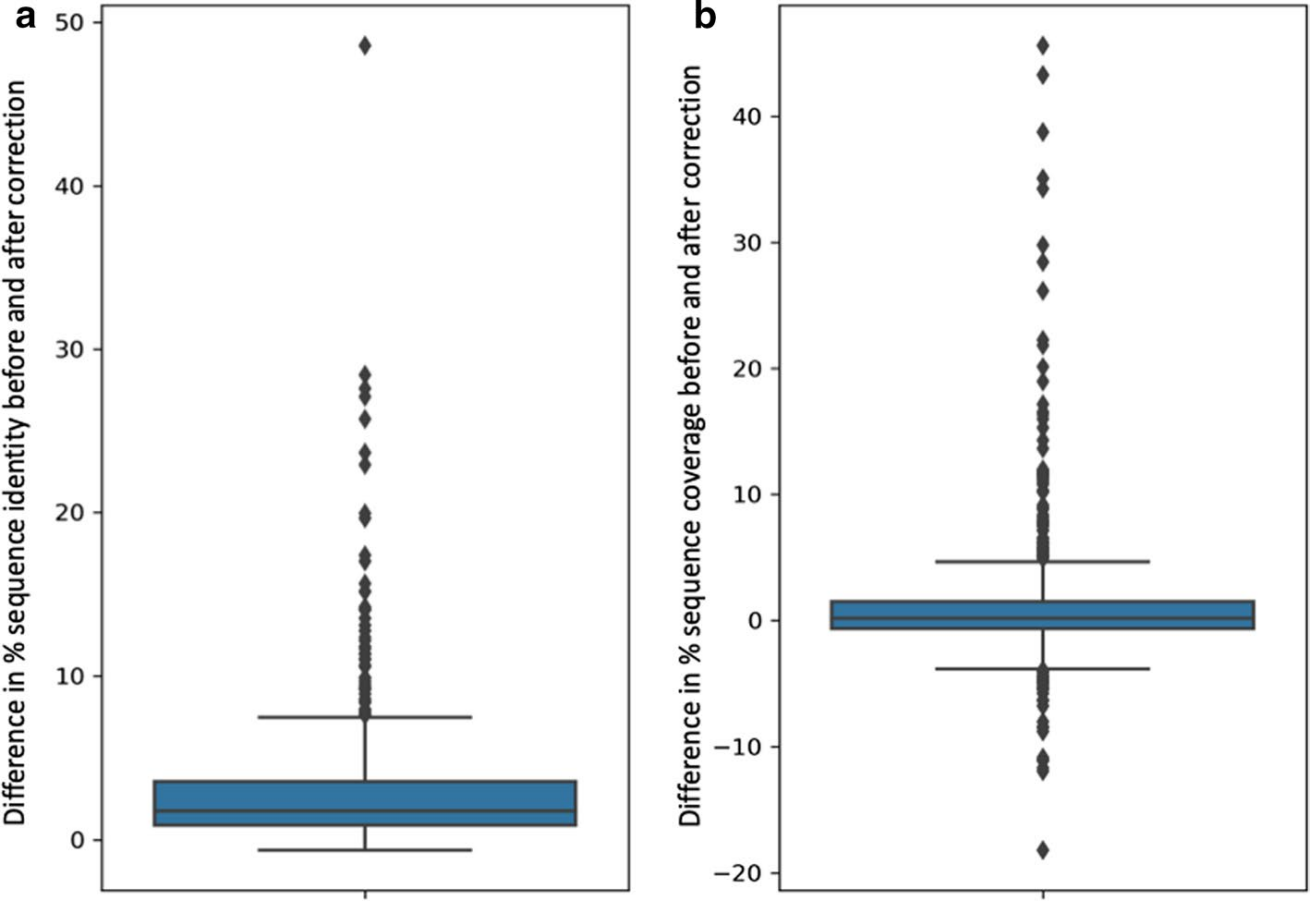
Boxplots of the difference before and after correction for **a** percent sequence identity and **b** percent sequence coverage, for each of the 603 primate protein sequences

We then estimated the quality of the refined primate sequences, by calculating the percent sequence identity and coverage for the original and refined primate sequences, compared to the human reference. On average, a small increase in both scores is observed after refinement, of + 3.1% and + 1.2% respectively (Table 4). However, the differences between the scores before and after correction between are not the same for the 603 sequences, as shown in Fig. 6. Some sequences had an increase in % sequence identity of > 50% and in coverage of > 45%, indicating the correction of very long mismatch errors. In contrast, a decrease in coverage was observed for a small number of sequences. This is probably be due to over-alignment of the mismatch-containing sequence, since the mismatched segment is aligned to the human reference even when it is not homologous, artificially boosting the original coverage score.

For most of the primates, higher quality sequences were proposed for between 20 and 80 proteins, while for *N. leucogenys*, 262 sequences were improved, representing 43% of the 603 corrections. This is not surprising since this primate is closely related to humans, and had the highest percentage of sequences with mismatch errors (Fig. 4).

## Discussion

DNA and protein sequence databases are increasingly useful research tools, but to maximize their potential, the errors in them need to be addressed. Contrary to expectations, advances in genome sequencing technologies have led to an increased number of protein sequence errors in public databases, including incomplete, incorrect or inconsistent sequences. Here, we used MSAs of closely related sequences to detect potential errors in primate proteins and investigate the underlying causes.

We used a simple protocol based on BLASTP searches (using very strict thresholds to limit the number of false positives) to identify potential orthologs of human proteins in ten primate proteomes. This method was chosen because it is time-efficient, convenient and allows identification of sequences in the most recent protein databases. More accurate tools for orthology detection have been developed, such as eggNOG [31], OrthoInspector [32], or OrthoFinder [33]. However, these methods are computationally costly and are not always updated with each release of the Uniprot, Ensembl or RefSeq databases. The strict thresholds used in our protocol mean that we eliminated many primate orthologs, for example for *Otolemur garnettii*, orthologs were identified for only 69% of the human reference sequences (Table 1). In the future, it would be useful to incorporate other methods, such as Reciprocal Blast Hits, in order to extend the method to allow analysis of more orthologs, as well as to other groups of organisms. Nevertheless, it is important to point out that our protocol relies on the presence of a well-studied organism with characterized proteins that can be used as a reference.

We then chose to focus our analyses on the sequence errors involving mismatched segments, since mismatch errors can have a serious effect on downstream analyses. Mismatched segments generally arise when an exon is translated in the wrong reading frame, or the exon boundaries are badly predicted and a non-coding nucleotide sequence is translated instead. To ensure that the mismatched segments detected by our protocol were indeed caused by such errors in gene prediction, we eliminated the mismatch errors that were associated with the possible identification of primate paralogs instead of orthologs, or misalignments during the MSA construction process. This resulted in a total of 11,015 mismatch errors in Uniprot protein sequences, and 5971 mismatch errors in RefSeq proteins. The difference in the number of mismatches can be partly explained by the fact that, in the Uniprot reference proteomes only one ‘canonical’ protein is provided for each gene, while in RefSeq all protein isoforms are listed, reducing the number of false positive mismatches due to the definition of alternative isoforms (with different exon–intron structures) for different species.

By mapping the Uniprot proteins to their genomic sequences in the Ensembl database, we investigated the underlying reasons for the predicted errors. Unfortunately, the extraction of the same information for the RefSeq sequences could not be fully automated, but we intend to address this issue in the future. Almost half (47.7%) of the 11,015 mismatch errors in the Uniprot sequences could be attributed to the presence of undetermined regions in the genomic sequences (represented by ‘N’ characters), causing misprediction of the gene exon–intron structures. Surprisingly, the undetermined region was not always located within the mismatched exon, and in these cases our error correction protocol was able to identify the correct exon sequence and improve the quality of the translated protein sequence.

Many of the remaining mismatch errors could be linked to other issues in the primate genome sequencing and/or assembly processes. For example, a large number of primate proteins (937) had abnormal introns of length < 30 nucleotides. In Human, it has been suggested previously that the minimal length for an intron to be processed is 30 nucleotides [30]. A number of mismatches were also linked to the presence of insertions or deletions of a small number of nucleotides (non-multiple of 3) within the exon sequence, meaning that part of the protein was predicted in the wrong frame or several short exons were predicted corresponding to one human exon. These results taken together clearly suggest that more robust gene prediction algorithms are needed that incorporate strategies for genome error detection or quality control.

Finally, a number of mismatch errors (237) were associated with the presence of non-canonical splice sites in the human reference gene, suggesting that the gene model used in the original primate genome annotation was incomplete and might be improved by taking into account non-canonical splice sites.

To test whether strategies could be developed to correct gene prediction errors, we implemented a simple error correction protocol as a proof-of-concept. A total of 603 protein sequences were refined and led to a significant reduction in the number of sequence errors, from 2184 errors before correction to 1379 errors after correction. The protocol involves the insertion of new exons in the primate transcripts, although the new exons are not guaranteed to have canonical splicing sites. This was done in order to produce a more accurate protein sequence, at the expense of transcript logic where splicing rules are not respected. This is only a first step in the improvement of gene prediction algorithms, but it demonstrates that the available primate genomes could be exploited to correct the primate proteins in public sequence databases.

## Conclusions

To conclude, by comparing ten primate proteomes with the human reference proteome, we showed that mismatch errors are frequent in primate proteomes, where the percentage of sequences having a mismatch error ranges from 2 to 12% depending on the primate species. Based on the features we identified, the reasons causing two thirds of these mismatches could be identified. Half of all potential mismatches were associated with evidence of gene misprediction. Interestingly, we showed that the existing primate genomes contain signals allowing improvement of these sequences.

The error detection protocol used here could be improved by the addition of new characterization features to understand the reasons for the detected mismatch errors that were not explained in our analyses, and to highlight the unexplained errors that may in fact be biological deviations representing organism specificities or innovations. It would also be interesting to extend our quality control protocol to other misprediction error types, such as insertions or deletions, as well as to the study of genomes from other species.

We hope that the results of this work will provide useful information for the development of more reliable gene prediction algorithms, as well as for downstream analyses that rely on high quality protein sequences. For example, the analysis protocol is currently being integrated in a new variant prediction system, called MISTIC [34], which uses the information in MSAs to predict the pathogenicity of human genome variants.

## Methods

### Human reference protein sequences

Human protein sequences are well studied and for the purposes of this study, are assumed to be accurate. The *Homo sapiens* reference proteome (UP000005640) from Uniprot (downloaded in Dec. 2019) was used for the reference protein sequences and we selected the canonical isoform for each human gene. A total of 20,596 human protein sequences, annotated as ‘Reviewed’ in the Uniprot database, were extracted.

### Orthologous sequences from primate proteomes

Potential orthology relationships between human proteins and all (Reviewed and Unreviewed) proteins from ten primates (*Pan Troglodytes, Gorilla gorilla gorilla, Macaca mulatta, Macaca fascicularis, Chlorocebus Sabaeus, Papio Anubis, Pongo abelii, Nomascus leucogenys, Callithrix jacchus, Otolemur garnettii*) were predicted using a simple protocol based on BLASTP [35] searches in the Uniprot reference proteome database. For each human protein sequence, the best hit in each of the ten primates was considered as an orthologous protein if it has an e-value < 10^–50^ and sequence identity > 80%. Table 1 shows details of the primate proteomes used and the number of predicted orthologous sequences.

Orthologous sequences were also identified using the same protocol in the proteomes corresponding to the ten primates in the RefSeq database (release 98). In this case, protein isoforms of the same gene are not grouped in the same database entry, we retained all protein sequences identified in the BLASTP searches with the same thresholds as above, i.e. an e-value < 10^–50^ and sequence identity > 80%.

### Multiple alignments and detection of sequence errors

For each of the 20,596 human proteins, a multiple sequence alignment (MSA) of the primate orthologous sequences was constructed using the MAFFT program [36]. Each of MSA was analyzed to detect potential insertions, deletions and mismatches in the orthologous sequences using the SIBIS program [37]. SIBIS uses a Bayesian framework combined with Dirichlet mixture models to identify inconsistent sequence segments representing potential sequence errors. The Bayesian approach provides a strong theoretical foundation for modeling the amino acid frequencies found at a specific alignment position. SIBIS combines a prior distribution of amino acid probabilities, with observed amino acid frequencies at homologous positions within the related proteins, in order to calculate scores for new sequences. The predicted errors are classified into seven categories: N-terminal deletion, N-terminal extension, C-terminal deletion, C-terminal extension, internal deletion, internal insertion and mismatched segment.

For this study, we focused on potential sequence mismatches, where a mismatch was defined as a sequence segment that is at least 20 amino acids long with less than 50% sequence identity between the human reference and the primate sequences. In addition, to exclude false positive mismatch errors due to wrong ortholog detection or MSA errors, mismatching sequences in primates that were significantly longer or shorter than the human sequence (primate sequence length three times longer or shorter than human) were excluded.

### Phylogenetic trees

Phylogenetic relations between organisms were determined using the LifeMap tool [38] by extracting a subtree based on the list of organisms of interest. The subtree was visualized using iTOL [39] generate the tree figures.

### Intron–exon maps and genomic sequences

To perform more in-depth analysis of mismatch errors, Uniprot protein sequences were mapped to their transcript IDs in the Ensembl database (release 99) using the Uniprot Retrieve/ID mapping tool. For each transcript, the CDS and genomic sequence were retrieved, as well as intron and exon positions on the genomic sequence.

### Mismatch characterization

For each potential mismatch error detected by SIBIS, the mismatch sequence segment was located on the exon/intron map, CDS and genomic region. To identify the potential causes of the mismatch error, nine features were defined and classified into three categories (Gene misprediction, False positive, Undetermined) as follows:

Evidence of a Gene prediction error involves five possible features:

i A low quality genomic sequence, containing ‘N’ characters indicating undetermined or ambiguous nucleotides (IUPAC nomenclature) probably caused by genome sequencing errors or assembly gaps,
j Short introns in the primate sequence (< 30 nucleotides), since in Human, it has been shown previously that the minimal length for an intron to be processed is 30 nucleotides [30],
k A mismatch coded by a single exon in human compared to > 3 exons in the primate sequence,
l Non-canonical splice sites (not GT/AG) in the human mismatch sequence,
m The presence of a small nucleotide insertion in the primate exon sequence causing a frameshift.

Evidence that the detected mismatch is in fact a False positive (i.e. the mismatch is due to inaccuracies in the quality control protocol) involves three possible features:

i The existence of alternative human isoform in the Ensembl database that does not Generate a mismatch at that position,
j MSA alignment errors,
k Mismatches in a sequence region with repeats.

The mismatch was characterized as Undetermined if one feature was detected, namely that the same mismatch is identified in four or more primates in the MSA, or a mixture of Gene misprediction and False positive evidence was identified.

### Mismatch error correction protocol

For the mismatch errors classified as gene mispredictions, a simple protocol was implemented to try to improve the quality of the protein sequence. Given a primate protein sequence with a mismatch error compared to the human sequence, the error correction protocol involves four steps (outlined in Fig. 2) as follows:

1. Using the human sequence segment as a query, perform a TBLASTN search in the primate genomic region corresponding to the transcript.
2. If a hit is found with an e-value < 0.001 and sequence identity > 80%, it is inserted into the primate transcript as a new exon.
3. Verify the integrity of the new transcript, by checking for overlapping or inverted exons, frameshifts and stop codons in the inserted sequence.
4. Create a new CDS and protein sequence.

To estimate the quality of the new protein sequence, the modified primate sequence was realigned with the human reference protein. Two scores were used to evaluate the quality of the primate sequences:

(i) Percent sequence identity I was defined as *I* = *i/N* × *100*, where *i* is the number of identical amino acids in the alignment and *N* is the total number of non-gap positions in the alignment.
(ii) Percent sequence coverage C was defined as *C* = *N/N*_*tot*_ × *100*, where *N* is the number of non-gap positions in the alignment and *N*_*tot*_ is the total length of the alignment.

### Implementation

The analyses were performed using an Sqlite3 database, in-house bash and Python (version 2.7) scripts, and numpy (numpy.org), pandas (pandas.pydata.org) and matplotlib (matplotlib.org) Python libraries. The Biopython (biopython.org) package was used to perform pairwise alignments during the mismatch characterization step. To retrieve large amounts of data from the Ensembl database, the grequests (pypi.org) package was used to perform asynchronous application programming interface (API) requests. The Json (www.json.org) library was used to process Ensembl API output.

## Supplementary information

**Additional file 1:** Characterization of the 5446 mismatch errors associated with gene mispredictions.

## Data Availability

All sequences used in this study are available in the public Uniprot, RefSeq and Ensembl databases. The results of the characterization of the mismatch errors associated with gene mispredictions are available in Additional file 1.
